# *Marteilia refringens* and *Marteilia pararefringens* sp. nov. are distinct parasites of bivalves and have different European distributions

**DOI:** 10.1017/S003118201800063X

**Published:** 2018-06-11

**Authors:** R. Kerr, G. M. Ward, G. D. Stentiford, A. Alfjorden, S. Mortensen, J. P. Bignell, S. W. Feist, A. Villalba, M. J. Carballal, A. Cao, I. Arzul, D. Ryder, D. Bass

**Affiliations:** 1 Pathology and Microbial Systematics Theme, Centre for Environment, Fisheries and Aquaculture Science (Cefas), Weymouth Laboratory, Weymouth, Dorset DT4 8UB, UK; 2 Biosciences, College of Life and Environmental Sciences, Stocker Road, University of Exeter, Exeter EX4 4QD, UK; 3 Department of Life Sciences, The Natural History Museum, Cromwell Road, SW7 5BD, London, UK; 4 Division of fish, Department of animal health and antimicrobial strategies, National Veterinary Institute (SVA), Sweden; 5 Institute of Marine Research, PO. Box 1870, Nordnes, 5817 Bergen, Norway; 6 Centro de Investigacións Mariñas, Consellería do Mar da Xunta de Galicia, 36620 Vilanova de Arousa, Spain; 7 Department of Life Sciences, University of Alcalá, 28871 Alcalá de Henares, Spain; 8 Institut Français de Recherche pour l'Exploitation de la Mer (Ifremer), Laboratoire de Génétique et Pathologie des Mollusques Marins, Avenue de Mus de Loup, 17390 La Tremblade, France

**Keywords:** *Marteilia refringens*, *Marteilia pararefringens*, ITS1 rDNA, IGS rDNA, Paramyxida, Ascetosporea, *Mytilus edulis*, *Ostrea edulis*

## Abstract

*Marteilia refringens* causes marteiliosis in oysters, mussels and other bivalve molluscs. This parasite previously comprised two species, *M. refringens* and *Marteilia maurini*, which were synonymized in 2007 and subsequently referred to as *M. refringens* ‘O-type’ and ‘M-type’. O-type has caused mass mortalities of the flat oyster *Ostrea edulis*. We used high throughput sequencing and histology to intensively screen flat oysters and mussels (*Mytilus edulis*) from the UK, Sweden and Norway for infection by both types and to generate multi-gene datasets to clarify their genetic distinctiveness. Mussels from the UK, Norway and Sweden were more frequently polymerase chain reaction (PCR)-positive for M-type (75/849) than oysters (11/542). We did not detect O-type in any northern European samples, and no histology-confirmed *Marteilia*-infected oysters were found in the UK, Norway and Sweden, even where co-habiting mussels were infected by the M-type. The two genetic lineages within ‘*M. refringens*’ are robustly distinguishable at species level. We therefore formally define them as separate species: *M. refringens* (previously O-type) and *Marteilia pararefringens* sp. nov. (M-type). We designed and tested new *Marteilia*-specific PCR primers amplifying from the 3’ end of the 18S rRNA gene through to the 5.8S gene, which specifically amplified the target region from both tissue and environmental samples.

## Introduction

There is no universal species definition for micro-eukaryotes (Boenigk *et al*. 2012). Whether they are parasitic or free-living, phenotypic evolution occurs at different rates to changes in genes used as taxonomic markers. Consequently, an informative threshold in marker difference to distinguish species in one group often does not work for another (Boenigk *et al*. 2012). Further, the most frequently used marker gene for protists, the 18S rRNA gene is more suitable for determining phylogenetic placement than for species-level discrimination. Therefore in cases of closely related species, multiple lines of evidence are required to objectively distinguish them. This can involve multiple genetic markers and a suite of phenotypic and/or ecological characteristics (Boenigk *et al*. 2012; Stentiford *et al*. 2014; Bass *et al.* 2009). We use this approach to resolve a long-term vacillation in the taxonomic status of lineages in the *Marteilia refringens* (Rhizaria, Ascetosporea, Paramyxida) ‘complex’, with consequences for policy and trade decisions.

*Marteilia refringens* infects commercially important bivalve species including the flat oyster *Ostrea edulis* and mussels *Mytilus edulis* and *Mytilus galloprovincialis* (Grizel *et al.* 1974; Villalba *et al.* 1993; Robledo and Figueras, 1995; Le Roux *et al.* 2001; López-Flores *et al.* 2004; Novoa *et al.* 2005). It has been recorded in Europe from the northern French coast southwards to the Mediterranean Sea, plus Corsica, Italy (including Sardinia), Slovenia, Portugal, Croatia, Greece and Tunisia. *Marteilia refringens* has been responsible for recurrent mass mortalities of *O. edulis* in Europe over the last four decades (Grizel *et al.* 1974; Berthe *et al.* 2004) and has thus been recognized by both the World Organization for Animal Health (OIE) and the European Union (under EC Directive 2006/88) as a significant pathogen of bivalve molluscs (OIE, 2017).

There are currently four *Marteilia* species with sequence data available (Ward *et al.* 2016): *M. refringens*, *M. cochilla* infecting the cockle *Cerastoderma edule* in Spain (Carrasco *et al.* 2013; Villalba *et al.* 2014), *Marteilia octospora* infecting the razor shell *Solen marginatus* in Spain (Ruiz *et al.* 2016) and *M. sydneyi* infecting Sydney rock oysters *Saccostrea glomerata* (Kleeman *et al.* 2004). Other related *Marteilia* lineages are known from 18S rRNA gene sequence data but have not been described morphologically (Ward *et al.* 2016). Some of these may correspond to the unsequenced *Marteilia* species noted in a review by Berthe *et al.* (2004). A related species, *M. granula* (Itoh *et al.* 2014) from the Manila clam *Ruditapes philippinarum*, has been re-named *Eomarteilia granula* (Ward *et al*. 2016).

*Marteilia refringens* was formerly recognized as two species, apparently separated by ultrastructural characteristics and host specificity: *M. refringens* infecting oysters and *M. maurini* infecting mussels (Grizel *et al.* 1974; Perkins, 1976; Comps *et al.* 1981; Figueras and Montes, 1988). However, subsequent studies concluded that these ultrastructural characteristics were invalid to distinguish between them (Villalba *et al.* 1993; Longshaw *et al.* 2001). They could also not be separated based on 18S rDNA sequence differences (Le Roux *et al.* 1999; Berthe *et al.* 2000), although using a polymerase chain reaction restriction fragment length polymorphism (PCR-RFLP) approach and sequencing of the more quickly-evolving ITS1 rDNA region, Le Roux *et al.* (2001) identified 40 ITS1 positions that were polymorphic between *M. refringens* infecting predominantly oysters (referred to as ‘O-type’) and mussels (‘M-type’).

However, host specificity was later shown also to be an unsuitable character for discriminating the two species. There is evidence for co-infections of ‘O’- and ‘M’-type *M. refringens* in the same host individual (Le Roux *et al.* 2001; López-Flores *et al.* 2004), and at one site (Huelva, Spain) studied by Novoa *et al.* (2005) 61% of M-type clones were isolated from oysters. Subsequent studies have reinforced these findings, although the degree of affinity of M-type to mussels and O-type to oysters has not been analysed. Furthermore, the host range of *M. refringens* is clearly wider than *O. edulis* and *M. edulis*: both types being detected in *Mytilus galloprovincialis* (Novoa *et al.* 2005; Balseiro *et al.* 2007) and the M-type in clam *Solen marginatus* (López-Flores *et al.* 2008*b*) and the mussel *Xenostrobus securis* (Pascual *et al.* 2010) and, the O-type in the clam *Chamelea gallina* (López-Flores *et al.* 2008*a*). Berthe *et al.* (2004) point out that unidentified *Marteilia* isolates have also been observed in many bivalve species naturally present in the geographic range of *M. refringens*.

The Pr4-Pr5 primer pair presented in Le Roux *et al.* (2001) has subsequently been used in several studies to generate more *M. refringens* ITS1 sequences from a range of hosts and to provide further evidence that *M. refringens* comprises two distinct genetic lineages (López-Flores *et al.* 2004; Novoa *et al.* 2005; Balseiro *et al.* 2007; Elgharsalli *et al.* 2013; Arzul *et al.* 2014; Gombac *et al.* 2014). This bipartition is also shown by IGS rDNA analyses (López-Flores *et al.* 2004, 2008*a*, *b*; Pascual *et al.* 2010; Elgharsalli *et al.* 2013). However, these rRNA gene/spacer sequence data have not been used to separate the types at species level; indeed, based on an analysis of the intergenic distances of a 358 bp region of *M. refringens* IGS sequences from mussels and oysters López-Flores *et al.* (2004) concluded that distances found were too small to constitute different species and the two types should be considered conspecific and therefore synonymous, a stance also taken by Balseiro *et al.* (2007). A working panel of the European Food Safety Authority (EFSA) on Animal Health and Welfare regarding the susceptibility to certain mollusc diseases concluded that *M. refringens* and *M. maurini* were synonymous (EFSA, 2007).

The synonymization of *M. refringens* and *M. maurini* had an important consequence: any new discovery of infection by either type could only be reported as *M. refringens,* a notifiable pathogen to both the OIE, and to the European Commission under Directive EC/2006/88. Subsequently, several sites in northern Europe (including in the UK (Tamar estuary on the English Channel coast), Sweden (NW coast) and Norway (Bømlo)) have been declared positive for *M. refringens*. In all three countries, no significant mortalities of mussels have occurred, and oysters have never been found to harbour *M. refringens* (of either type) (indirectly reported for the UK in Laing *et al.* 2014).

As there have been no verified reports of O-type *M. refringens* from northern Europe we sequenced ITS rDNA regions from as many examples of the parasite as were available from this region to determine whether they were O- or M-type, and integrated these results with the findings of a comprehensive literature survey to summarize the geographical distribution of each. We sequenced the full-length ribosomal RNA gene arrays from O- and M-type lineages to assess the relative reliability of sequence differences (sequence signatures) across this region for distinguishing them based on gene sequences alone. The identification of the most promising diagnostic sites allowed the design of a new primer set that could be used for future typing and eDNA studies. In combination, our findings provide a sufficiently strong basis for reinstating two separate species, which we formally carry out in this paper. Given that we propose taxonomic distinction of O- and M- types based upon phylogenetic data presented herein and we offer a means of discriminating these taxa based upon a specific diagnostic, our study underpins a basis for updating the listing of *M. refringens* as a notifiable disease of molluscs in current OIE and EC legislation.

## Materials and methods

### Histology

Excised digestive gland and mantle samples from oysters and mussels were placed immediately into Davidson's seawater fixative and fixation allowed to proceed for 24 h before transfer to 70% industrial methylated spirit prior to processing. Fixed samples were processed to wax in a vacuum infiltration processor using standard protocols and 3–5 *µ*m sections were cut using a rotary microtome prior to mounting on glass slides and staining with haematoxylin and eosin (HE). Stained sections were analysed by light microscopy (Nikon Eclipse E800) and digital images were taken using the Lucia™ Screen Measurement System (Nikon, UK).

### Sample acquisition and DNA extraction

*Marteilia refringens*-infected samples of *Ostrea edulis*, *Mytilus edulis,* and *M. galloprovincialis* and *M. cochillia*-infected samples of *Cerastoderma edule* were obtained from sites in the UK, Spain, France, Sweden and Norway (Table 1). All of the mussel samples from the Tamar, Sweden and Norway were amplified with primers Me15–Me16 targeting the Glu (adhesive protein) gene as devised by Inoue *et al*. (1995) and used by Bignell *et al*. (2008), confirming that that mussels sampled from these countries were *M. edulis*, not *M. galloprovincialis*, or hybrids between the two. The materials from France, Spain and some of the UK samples were known/putative *Marteilia* positives and were used for generating rRNA gene amplicons to create or map to the rRNA gene array. These are indicated by grey text in Table 1. All other UK samples and those from Sweden and Norway were used for *Marteilia* screening by group-specific PCR and histology. There are no records to date of *M. provincialis* in Sweden (reviewed in Aku, 2018).

**Table 1.** Bivalve samples used to generate ITS1 and/or IGS rDNA sequences as part of this study

Host	Sampling information		Tissue type	Histology		Molecular analysis		Gene array region	Genotype	Sample ID
	Location	Date		No. screened	No. + ve	No. screened	No. + ve			
***Mytlius edulis***	Tamar estuary, UK	2012	DG, spores	N/A	N/A	N/A	N/A	ITS1, IGS	M	RA12041: 91–96 (DG); F1-10 (spores)
	Tamar estuary, UK	2012	DG	N/A	N/A	N/A	N/A	ITS1	M	(PM)23176: 22,29,84,131
	**Tamar estuary, UK**	**06/2013**	**DG**	**300**	**9**	**300**	**33**	**ITS1**	**M**	**RA13082(*n* = 150); RA13085(*n* = 150)**
	**Tamar estuary, UK**	**09/2015**	**DG, mantle**	**150**	**0**	**150**	**4**	**ITS1**	**M**	**RA15100/PM29721**
	**Tamar estuary, UK**	**09/2016**	**DG, mantle**	**75**	**0**	**75**	**0**	**ITS1**	**M**	**RA16043**
	**NW coast, Sweden**	**09/2014**	**DG**	**30**	**20**	**300**	**22**	**ITS1**	**M**	**Swe1-18**
	**Bømlo, W. Norway**	**07/2016**	**DG**	**30**	**13**	**24**	**16**	**ITS1**	**M**	**16/24:1-30**
*M. galloprovincialis*	Spain, Vigo	2012	DG	N/A	12	N/A	11	ITS1	M	RA12041: 122-135
	Brest, France	2012	DG	N/A	2	N/A	2	ITS1	M	08/54/41/1, 08/54/46/2
***Ostrea edulis***	**Tamar estuary, UK**	**09/2015**	**DG**	**212**	**0**	**212**	**0**	**ITS1**	**M**	**RA15100/PM29721**
	**Tamar estuary, UK**	**09/2016**	**DG**	**150**	**0**	**150**	**1**	**ITS1**	**M**	**PM31823**
	Brest, France	2012	DG	N/A	N/A	N/A	2	ITS1, IGS	O	07/47/17, 07/47/20, fo1-5
	**NW coast, Sweden**	**09/2014**	**DG**	**0**	**0**	**150**	**0**	**ITS1**	**M**	**n/a**
	**Bømlo, W. Norway**	**07/2016**	**DG**	**30**	**0**	**30**	**10**	**ITS1**	**M**	**16/21:1-30**
*Cerastoderma edule*	Spain, Vigo	2012	DG	N/A	10	N/A	10	ITS1	*M. cochillia*	RA12041: 111-120

Grey text indicates known/putatively infected reference material used to generate amplicons and metagenomic sequence libraries for rRNA gene array assemblies (18S-ITS1-5.8S-ITS2-28S-ISG), not for screening for presence of *Marteilia*.

Tissue samples (digestive gland (DG) and mantle; Table 1) were homogenized using a Fastprep 24 homogeniser and Lysing Matrix A tubes (MP Biomedicals). *Marteilia* sporangia (10 samples obtained from infected UK *M. edulis* samples RA12041: 91–96) were purified following the method of Robledo *et al.* (1995) but using a 100 *µ*m mesh to sieve the homogenate prior to separation using a sucrose gradient. DNA was extracted from 5 mg of each tissue or 50 *µ*L purified sporangia homogenate using the EZ1 Advanced XL Biorobot and DNA Tissue extraction kit (Qiagen) after Proteinase K digestion at 56 °C for 4 h. Separate UK samples (RA15100 1-22) and 20 histology-positive samples collected from Swedish mussels were extracted using phenol/chloroform (Nishiguchi *et al*. 2002). DNA was extracted from 24 Norwegian mussel (*M*. *edulis*) and 30 oyster (*O. edulis*) samples using QIAamp DNA mini kit (Qiagen), after Proteinase K digestion at 56 °C overnight. All DNA was quantified and checked for purity using Nanodrop (Thermo Scientific) and QuantiFluor^®^ dsDNA System on the Quantus^™^ Fluorometer. Filtered water, sediment samples and potential alternative invertebrate hosts from the Tamar Estuary were collected and processed as described in Ward *et al.* (2016). Other invertebrate samples collected as part of those studies were also screened with new *Marteilia*-specific primers designed during the present study (see below).

### Metagenomic sequencing: ribosomal RNA gene array sequencing and assembly

Three pools of DNA were constructed from four of the 10 sporangia isolated from UK *M. edulis*, infected digestive glands of *M. galloprovincialis* from France (08/54/41/1 & 08/54/46/2) and digestive glands of *C. edule* from Spain infected with *M. cochillia* (RA12041: 111–120) (Table 1). These were prepared for metagenomic sequencing using the Illumina compatible NEXTflex^™^ PCR-Free DNA Sequencing Kit (Newmarket Scientific, 2 × 300 bp paired-end reads) and sequenced on a MiSeq 300 at the University of Exeter, UK. Raw sequences were processed as follows: Adaptor sequences were trimmed using Trimmomatic 0.32 (Bolger *et al.* 2014). Prinseq Lite (Schmieder and Edwards, 2011) was used for more stringent quality filtering, removing reads containing ambiguous bases or where the mean quality of the reads fell below 25. The 3’ end of reads were trimmed where the quality of bases fell below 25. FastQC (Andrews *et al.* http://www.bioinformatics.babraham.ac.uk/projects/fastqc/) was used to check the quality of reads, read pairs from each library were aligned and merged using FLASH (Magoč and Salzberg, 2011). A minimum overlap of 10 base pairs and a maximum mismatch density of 0.25 was set, together with an estimated read and fragment length of 300 and 600 base pairs, respectively. After merging paired-end reads, sequences from each library were converted into BLAST databases, against which *M. refringens* 28S rDNA sequence AJ604561 was locally blasted to retrieve seed regions for rDNA array assembly using MITObim version 1.6 (Hahn *et al.* 2013).

### Amplicon generation and sequencing

**Fig. 1.**
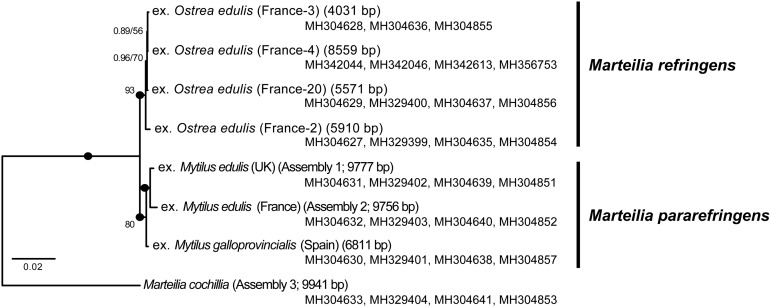
Bayesian phylogeny of the three full length rRNA gene array assemblies (1–3; two from *Mytilus edulis* infected with M-type *Marteilia refringens* from the UK and France, and *Cerastoderma edule* from Spain infected with *Marteilia cochillia*) with the longest incomplete array sequences (all >4 kbp) generated from O-type infections of *Ostrea edulis* from France and *Mytilus galloprovincialis* from Spain (M-type). Numbers of positions of each sequence are given in brackets. 10 096 positions were analysed; incomplete sequences were padded with missing data points. Bayesian posterior probability (BPP) supports and Maximum Likelihood bootstrap supports are shown at each node. Blobs indicate BPP = 1.0.

**Fig. 2.**
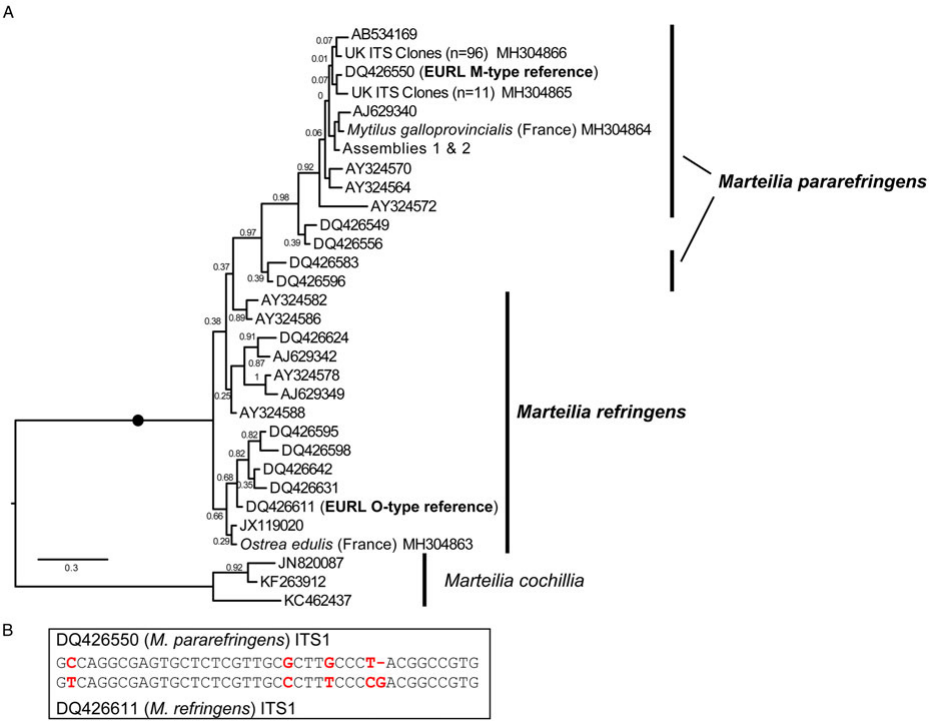
A: Maximum Likelihood (ML) phylogeny of ITS1 rDNA sequence types from GenBank and generated by this study. 336 positions were analysed. 302 ITS1 reads were reduced to the 31 genotypes represented in this tree by not including identical reads and minor-variant singleton sequences in the analysis. Maximum Likelihood bootstrap supports are shown at each node. Blobs indicate BPP = 1.0. The tree is rooted on *M. cochillia*. B: region of ITS1 rDNA containing the five positions distinguishing O- and M-types *M. refringens* (i.e. *M. refringens* and *M. pararefringens*), which are invariant within each type/species.

**Fig. 3.**
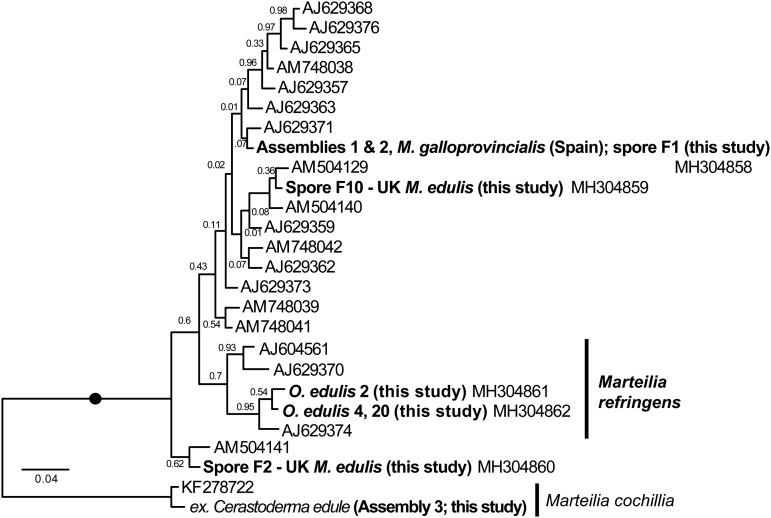
Maximum Likelihood (ML) phylogeny of IGS rDNA sequence types from GenBank and generated by this study. 366 positions were analysed. 68 IGS reads were reduced to the 27 sequences represented in this tree by not including identical reads and minor-variant singleton sequences in the analysis. Maximum Likelihood bootstrap supports are shown at each node. Blobs indicate BPP = 1.0. The tree is rooted on *M. cochillia*. O-type (*Marteilia refringens*) sequences are labeled in the larger clade; all other sequences are (paraphyletic) M-type (*M. pararefringens*).

The three rRNA gene array (18S-ITS1-5.8S-ITS2-28S-ISG) assemblies were aligned using MAFFT (Katoh and Standley, 2013). The alignment was then used to design primers to amplify both *M. refringens* types but not *M. cochillia* across the c. 10.5 kb rDNA alignment (Table S1). Using these primers, the previously published ITS1 primers Pr4-Pr5 (Le Roux *et al.* 2001), and the nested IGS primer set MT-1/MT-2 & MT-1B/MT-2B (López-Flores *et al.* 2004), partial but substantial coverage of the array was obtained for six French oysters (total lengths 8559, 5910, 5571, 4031, 1817 and 1722 bpp), French mussels #08/54/46 (1816 bp), Spanish mussels RA12041:124 (1805 bp) and 125 (6811 bp) and sporangia from UK mussels (2 × 1816 bpp). Samples for which total sequence lengths of >4000 bp were obtained were used to generate Fig. 1. PCR reactions were performed in 50 *μ*L reactions consisting of 10 *μ*L 5X Green Go Taq buffer (Promega), 5 *μ*L 25 mm MgCl_2_, 0.5 *μ*L 25 mm dNTPs, 0.5 *μ*L of each of 100 *μ*m forward and reverse primer, 1.25 units Go Taq Flexi (Promega), 2.5 *μ*L DNA, and 30.75 *μ*L H_2_O. Amplifications were performed on a Peltier PTC-225 thermal cycler using the following program: 94 °C × 5 min followed by 40 cycles of 94 °C × 1 min, *°C × 1 min and 72 °C × 1 min, followed by 72 °C × 10 min and held at 4 °C. The Norwegian samples only were PCRed as follows (according to local diagnostic laboratory practice): 50 *µ*L reactions comprising 1X PCR buffer (Qiagen), 1 × Q solution, 50 pmol primer solution, 0,2 mm dNTPs, 1.25 U HotStarTaq polymerase, 100 ng DNA template. Annealing temperatures for each new primer set used are as detailed in Table S1. Pr4/Pr5 and MT-1/MT-2 used an annealing temperature of 55 °C (60 °C in Norway). The second round PCR of the IGS (MT-1B/MT-2B) used thermal cycling conditions of: 94 °C × 5 m, followed by 25 cycles of 94 °C × 30 s, 60 °C × 30 s, 72 °C × 30 s followed by 72 °C × 5 min and held at 4 °C. Amplification products were resolved on 2% agarose gels stained with ethidium bromide and visualized using a UV illuminator. Correct size products were excised from the gels, purified using the Wizard SV gel and PCR purification system (Promega) and sequenced using the ABI PRISIM Big Dye Terminator v3.1 cycle sequencing kit following manufacturer's instructions. DNA was sequenced using the ABI 3130xl Avant Genetic analyser (Applied Biosystems). Analysis of the sequences was completed using Sequencher software (Gene codes corporation). Newly generated sequences analysed in this study are available from GenBank as shown in Figs 1–3.

### Signature sequence and phylogenetic analyses

The sequences acquired by targeted PCR were aligned to the longer, assembled sequences using MAFFT (Katoh and Standley, 2013). Additionally, all *M. refringens*-like ITS1 and IGS sequences available on NCBI Genbank as of October 2015 (Tables S2 and S3; ITS1 from López-Flores *et al.* 2004; Novoa *et al.* 2005; Balserio *et al.* 2007; Elgharsalli *et al.* 2013; Arzul *et al.* 2014; Gombac *et al.* 2014; all generated using primers Pr4-Pr5; IGS from López-Flores *et al.* 2004, 2008*a*, *b*; Carrasco *et al.* 2007*a*,*b*; Pascual *et al.* 2010; Elgharsalli *et al.* 2013) were downloaded and added to this alignment, which was then scrutinized by eye to identify putative signature sequences corresponding to O- and M-type sequences based on the EURL reference strain ITS sequences DQ426611 and DQ426550 respectively, and IGS sequences from the literature. Array regions without a minimum of two of each sequence type were ignored for this process.

Phylogenetic analyses were carried out on the alignment of >4000 bp rRNA (partial) gene array sequences (Fig. 1), and on separate ITS and IGS alignments (Figs 2 & 3). Bayesian consensus trees were constructed using MrBayes v 3.2 (Ronquist *et al*. (2012) in parallel mode (Altekar *et al.* 2004) on the Cipres Science Gateway (Miller *et al.* 2010). Two separate MC^3^ runs with randomly generated starting trees were carried out for 4 million generations each with one cold and three heated chains. The evolutionary model applied included a GTR substitution matrix, a four-category autocorrelated gamma correction and the covarion model. All parameters were estimated from the data. Trees were sampled every 100 generations. One-million generations were discarded as ‘burn-in’ (trees sampled before the likelihood plots reached a plateau) and a consensus tree was constructed from the remaining sample. Bootstrapped Maximum Likelihood (ML) trees were then calculated via the Cipres Science Gateway server (Miller *et al.* 2010) using RAxML BlackBox version 8.2.9 (Stamatakis 2014; Stamatakis *et al.* 2008) (GTR + CAT; all parameters estimated from the data); bootstrap values were mapped onto the highest likelihood tree obtained.

### High throughput sequencing and analyses of ITS1 amplicons

The primers Pr4-Pr5 (Le Roux *et al.* 2001) were used as previously described to screen a total of 362 *O. edulis* and 525 *M. edulis* digestive gland/mantle samples taken between 2013 and 2016 from the Tamar estuary, UK, 300 *M. edulis* from the NW coast of Sweden and 24 from Bømlo, western Norway. The same primers were used to amplify *M. refringens* ITS sequences from samples of spores (*n* = 6) and histology-positive *M. edulis* from Sweden (*n* = 20).

The 2013 Cremyll Ferry and the 2015 Swedish samples were also screened using a new nested primer set designed for this study (MartDBITS F1R1/F2R2). The primer set was designed to amplify from the V9 region of the 18S to the 5.8S (product size c. 1034 bp) covering the entire ITS1 region (Table S1). The target lineages were those with 18S sequences identical or very similar to *M. refringens*, including *M. cochillia* and *M. octospora*. Thermocyling conditions were the same as described for Pr4/Pr5 but using a 65 °C annealing temperature, 35 cycles and an extension time of 7 min. Positive samples from this screen were purified from a gel using spin module and recovery tubes (MP Biomedicals).

ITS amplicons from all *M. edulis* tissue samples from the UK and Sweden positive for *M. refringens* by histology and/or lineage-specific PCR were pooled into six batches (TAM-1, TAM-2, TAM-3, TAM-4, SWE-1, SWE-2) as shown in Table 2. The DNA content of samples comprising each pool was equalized. For each pool, a sequencing library was constructed using the TruSeq DNA PCR-Free Sample Preparation Kit (Illumina). The TruSeq Nano DNA Sample Preparation Kit (Illumina) was used for TAM-2, which had too little DNA for a PCR-free prep. The libraries were sequenced on an Illumina MiSeq at the Natural History Museum, London.

**Table 2.** Composition of ITS1 amplicon libraries sequenced by Illumina MiSeq.

Mussel Batch	Sample Type	Site	Primers	Miseq Library (number of OTUs)
Tamar 2012	DNA from spores isolated from infected mussels (*n* = 6)	Cremyll Ferry,Tamar	Pr4-5	TAM-1
Tamar 2013–A	DNA and cDNA from PCR + ve, histology –ve mussels (*n* = 33)	Cremyll Ferry,Tamar	Pr4-5	TAM-2
			MartDBITS	TAM-3
Tamar 2013–B	DNA and cDNA from PCR + ve, histology + ve mussels (*n* = 9)	Cremyll Ferry,Tamar	Pr4-5	TAM-2
			MartDBITS	TAM-4
Tamar 2015	DNA from PCR + ve mussels (*n* = 4)	Jupiter Point, Tamar	Pr4-5	TAM-2
Sweden 2015	DNA from diseased (histology + ve) mussels (*n* = 20)	Gothenburg, Sweden	Pr4-5	SWE-1
			MartDBITS	SWE-2

The raw amplicon sequences were processed using Prinseq Lite, FastQC and FLASH as described above. Merged paired sequences containing a quality score of 15 or less were removed as were those longer than 250 bp. Sequences were dereplicated, sorted by size and clustered into operational taxonomic units (OTUs) using a 1% difference between clusters (to ensure that O- and M-types would both be detected if present, based on the five invariant positional differences). The OTUs were aligned using MAFFT to the EURL reference sequences DQ426611 (O-type) and DQ426550 (M-type); Arzul *et al.* 2014). Sequences were then scrutinized in Bioedit (Hall, 1999) by eye using the signatures to determine the genotype(s) present.

## Results

Complete 18S-ITS1-5.8S-ITS2-28S-IGS ribosomal gene arrays for *M. refringens* were generated and aligned from spores isolated from UK *M. edulis* (Assembly 1, 9777 bp; M-type), French *M. galloprovincialis* digestive gland tissue (Assembly 2, 9756 bp; M-type) and *M. cochillia*-infected digestive gland tissue from *Cerastoderma edule* from Spain (Assembly 3, 9941 bp).

After mapping amplicons from GenBank and those generated by this study to the array assembly alignment, regions where two or more sequences each derived from independent O- and M-type samples were present (which excluded almost all of the 28S) were examined for O/M-type signatures. We identified 32 candidate signatures (substitutions and indels) that show the division of the samples into O- and M-type: 11 in ITS1, 12 in ITS2, one in 28S and eight in IGS (Table S4). The ITS1 and IGS regions were the most highly represented by sequences in GenBank, so we focused on these. Only five sites in the ITS1 region were invariably in one of two configurations (Fig. 2B) that corresponded with the two EURL reference sequences and agreed with the bipartition of strains reported by Le Roux *et al.* (2001). There was a more variable region at the 3′ end of ITS2 that may prove to be an equally good set of markers for the two types, but more samples are required to confirm this; similarly for other, more isolated SNP-like sites, elsewhere in the array. However, some sites in ITS1 and IGS regions, although largely consistent within one or other of the lineages, had different nucleotides in some of the samples and are therefore not reliable markers of O- *vs* M-types. Detailed information about all of these sites is given in Table S4.

This pattern of variation is reflected in phylogenetic analyses based on the different array regions. A Bayesian phylogenetic tree based on the longest sequences across the whole rDNA array shows that the two genotypes form separate clades, each with full Bayesian Posterior Probability support (Fig. 1). However, phylogenetic analyses separately based on only the ITS1 and IGS regions recover monophyletic type-specific clades only weakly, or not at all (Figs 2A & 3). These regions are short and carry very little phylogenetic signal.

We aligned 302 ITS1 region sequences (101 O-type, 201 M-type) from this study and downloaded from GenBank and then reduced this to a de-duplicated alignment of 31 sequences, representing the full breadth of known diversity. In the resulting Bayesian tree (Fig. 2A) neither O- or M-type clade is monophyletic. In many cases, this is caused by conflicting signal, e.g. O-type sequences DQ426549 and DQ426556 group with the M-type clade in Fig. 2A because sequence positions outside of the five invariant sites have mixed O/M-type characters. The tree also shows significant sequence diversity within the two main types, the significance of which is unknown. All of the within-lineage diversity shown in Fig. 2A was detected in at least two independent samples (individuals). Most of the branches are represented by several independent samples, indicating that these variants represent true micro-variation between lineages. An IGS alignment of sequences downloaded from GenBank and generated by this study shows a comparable pattern: a monophyletic but relatively weakly supported O-type clade within a paraphyletic M-type (Fig. 3).

*Mytilus* spp and *O. edulis* individuals from the UK, Sweden, Norway, France and Spain that were *M. refringens*-positive by histology were PCR-amplified using the EURL diagnostic ITS1 primers Pr4/Pr5 and in some cases also with our new ITS1 primers (this study), or IGS primers (López-Flores *et al.* 2004). Some UK sample sets were screened in full by both molecular and histological methods (Table 1). The results were striking: only M-type was detected in the northernmost countries UK, Sweden and Norway (see Table 1). Further, infections were only found in mussels in these countries, never oysters. All stages from primary cells to mature spores were observed in multiple individuals from each country, although not all infections exhibited intense infections and/or sporogonic stages. Oyster DG samples from the UK, Sweden and Norway (Table 1) were PCR-screened (EURL and new ITS1 primers) but were mostly negative (1/512 *O. edulis* samples from the UK and Sweden (2014–16); Table 1), although in all cases the oysters sampled were growing in close, often physical, contact with infected mussels. The highest proportion of PCR-positive oysters (10/30; Table 1) were sampled from the Aga oyster poll (lagoon), Norway, in which the cohabiting mussels showed high levels of infection by M-type (13/30 individuals by histology; Table 1), which additionally were sporulating in the lagoon at the time of sampling. None of the PCR-positive oysters were histology positive for *Marteilia*. On the other hand, our literature survey showed that, where ITS1 sequences were available, both genotypes have been detected in central and southern European countries and that each infected both oysters and mussels (Tables S2 & S3).

To ascertain whether the O-type was present in the UK and Swedish samples even at very low levels, we pooled ITS1 amplicons from all infected individual mussels available from our sampling in these two countries and deeply sequenced these on an Illumina MiSeq (Table 2). Only M-type sequences were recovered, as determined by the five invariant signatures described above (Fig. 2). (The Norwegian samples were not available at the time of MiSeq sequencing.)

Our new nested diagnostic primers (MartDBITS, Table S1) amplify a 1034 bp region from the 3′ end of the 18S rRNA gene V9 region through to the 5.8S rRNA gene, therefore spanning the whole of ITS1. Where the same samples were also amplified with Pr4/5 the nested primers often gave much stronger and clearer products, and worked consistently on all samples shown to be positive using the Pr4/Pr5 primer set. The nested primers also consistently worked on environmental samples (filtered water, sediment, etc.; Table 3), whereas Pr4/5 generally did not. We detected *M. pararefringens* (only) from filtered water samples (in two size fractions) and sediments sampled from near mussel/oyster beds in the Tamar Estuary, UK, in 2013–2016, but not 2017, the only year in which no histology- or PCR-positive animals were found at that site. The amplicons generated by these primers allow discrimination between *M*. *refringens* and *M*. *pararefringens* via sequencing and should also amplify closely related *Marteilia* lineages. However, no other *Marteilia* genotypes were amplified during the course of this study. We screened a range of other potential alternative hosts from the Tamar and other UK sites (amphipods, copepods, isopods, barnacles, nudibranchs, lobster larvae, gastropods; 20 individuals of each); none of these were positive for any *Marteilia* genotype.

**Table 3.** Performance of *Marteilia*-specific 18S–5.8S rRNA gene primers designed in this study, on DNA samples from the Tamar estuary, 2013–2017

Year	0.45 *µ*m-filtered water	20 *µ*m-filtered water	Sediment
2013	4/25	1/7	1/2
2015	4/9	−	2/9
2016	3/9	−	−
2017	0/14	−	−

Bivalve DNA samples not reported: the new primers performed equivalently to Pr4-Pr5 in the subset of samples tested. *x*/*y* indicates the number of positive samples out of total tested. All positives were confirmed as *M. pararefringens* by sequencing. No non-target sequences were amplified. Dashes = no samples tested.

## Discussion

Robust discrimination between pathogenic species has important consequence for listing/trade in global animal commodities (Stentiford *et al*. 2014). ‘*Marteilia refringens*’ has become a vaguely defined taxon known to comprise more than one parasite lineage, but the status and nature of these lineages have become confused by geography, host and, pathogen morphological conservation. This confusion led to their listing as a single entity listing by EC/OIE, with stringent implications for trade. A growing awareness of the utility of molecular genetic data and analyses to define taxonomic boundaries at high resolution offers a solution to such ambiguities. Our combined analysis of multi-locus marker comparison and targeted sampling provides an illustration of the potential of this approach to make considered and objective taxonomic decisions, which can underpin listing decisions and form the basis of future research.

Previous studies (see Introduction) have shown that *M. refringens* M- and O-types group separately on phylogenetic trees and can be identified by certain sequence alignment characteristics in the ITS1 and IGS rDNA regions. However, phylogenetic trees based on short amplicons such as generated by the frequently used ITS1 and IGS primers are weakly resolved due to lack of (and sometimes conflicting) phylogenetic signal contained in those amplicons. We show in this study that when the total known sequence diversity of ITS1 and IGS regions is included in separate phylogenetic analyses, robust monophyly of M- and O-type clades is not recovered, whereas analyses based on the full rRNA gene array do achieve this. This finding has two consequences: (1) ‘O-’ and ‘M-type’ genotypes are confirmed as robustly mutually exclusive, and differ genetically at a level consistent with distinct species status in other eukaryote groups (Boenigk *et al*. 2012), but (2) assigning O/M genotypes using phylogenetic inference based on single marker genes can be unreliable.

A solution to the second point is to use sequence signatures as a diagnostic for each genotype. This approach has been used many times in protistology at species level and below (e.g. Wright *et al.* 1997; Amato *et al.* 2007; Bass *et al.* 2009; Rynearson *et al.* 2009; Nath *et al.* 2012) and at higher taxonomic levels (Cavalier-Smith and Chao, 2003; Karpov *et al.* 2006; Burki *et al.* 2010). Sequence signatures are also used in bacteriology and metazoan parasitology (Pettersson *et al.* 1996; Egyed *et al.* 2001; Tung *et al.* 2007). However, it is important that such signatures are reliable (invariable within types). Our meta-analysis of all available ITS1 and IGS *Marteilia* sequences identified a cluster of five signatures distinguishing *M. refringens* and *M. pararefringens* in the ITS1 rDNA that were absolutely invariant across all available sequences. These correspond to the positions shaded in grey on the lower section of Fig. 2 in Novoa *et al.* (2005) and boxed on Fig. 4 of Gombac *et al.* (2014). We therefore confirm and recommend these as the most reliably diagnostic signatures.

Our results also strongly suggest that *M. refringens* and *M. pararefringens* have different distributions. There is currently no evidence of *M. refringens* north of France. We sampled large numbers of *M. edulis* and *O. edulis* (in most cases from co-occurring populations) in the UK (Tamar estuary), Sweden, and Norway: only *M. pararefringens* was detected, infecting the mussels to varying degrees as confirmed by histology, but never the oysters. A larger number of mussel tissue samples were PCR-positive for *M. pararefringens* than were histology-positive, due to low-level infections (and in some cases possibly the presence of non-infective material). However, 10/30 and 1/362 oyster DG samples from the Norwegian lagoon and Tamar Estuary, respectively were PCR positive; all *M. pararefringens*, but no infection was seen by histological examination of all 11 PCR-positive samples. In the Norwegian oyster poll, *M. pararefringens* infection frequency of mussels was high, sporulation was occurring (therefore increasing the potential for passive uptake and contamination by spores), and the hydrographics of the lagoon act to concentrate material within it. Therefore we suggest that passive association of bivalve individuals with *M. pararefringens* material is generally rare, and was only the case here because of the high frequency of infected mussels. It is significant that even in this northern European habitat clearly conducive to *M. pararefringens* proliferation (a) only this species was present, and (b) infection was not recorded in oysters. At a late stage of writing of this paper an additional incidence of *M. pararefringens* infection in *M. edulis* was reported by the competent authority in Northern Ireland (communicated by EURL for molluscan diseases).

Consistent with these results are previous findings of a programme monitoring for presence of *Marteilia* and *Bonamia* in *O. edulis* running from 1982 to 2014 (Laing *et al.* 2014). In total 76 307 oysters from 144 sites in England and Wales were examined by histology, in which no cases of *Marteilia* infection were found. Similarly, routine sampling of 2,985 Swedish oysters in 1995–6 and from 2006–15 by the Swedish Veterinary Institute revealed no *Marteilia* infections (unpublished data), and oysters infected with *Marteilia* have also never been recorded in Norway.

We chose to study sites in the UK, Sweden and Norway where oysters and mussels were growing in contact with each other, and therefore theoretically exposed to the same potential pathogens. Supporting this theory, Ward *et al*. (2016) showed that at the UK site, *M. pararefringens* could be detected by eDNA methods in filtered water from directly above the oyster/mussel beds. Perhaps similarly, van Banning (1979) reported that Dutch healthy oysters in contact with French infected oysters did not become infected in the Dutch environment (October to December 1975 at 5–10 °C) or during co-occurrence of French and Dutch oysters at 15 °C in experimental aquaria.

Although our intensive sampling – including deep sequencing of amplicons from all infected material from the UK and Sweden available to us – did not detect *M. refringens*, we cannot exclude the possibility that it exists at low levels in these northern European sites. For free-living protists at least, very large or global distributions are frequent (Bass and Boenigk, 2011). Therefore if *M. refringens* is really absent (or at least effectively so) from some regions there should be a parasitological explanation.

*Marteilia refringens* is apparently temperature-dependent and infects other hosts as part of a complex lifecycle (Arzul *et al.* 2014). As we have evidence only of infection by *M. pararefringens* in northern Europe, it is possible that the climatic/ecological conditions are unsuitable for *M. refringens* to exist or complete its lifecycle, perhaps because of the absence of a suitable vector or other lifecycle requirement. A further question relates to the geographical distribution of conditions conducive to *M. pararefringens* clinical disease rather than just infection. In the UK, Sweden and Norway clinical disease in mussels has only been reported at the individual level without causing population-level epidemics (Bignell *et al*. 2011). So it is apparent that *M. pararefringens* can complete its lifecycle in *M. edulis* in northern Europe, though possibly not in other species. If *M. refringens* cannot complete its lifecycle in any host then it would not be able to establish.

A further interesting point is that even though both types can infect a range of hosts more southerly in Europe, studies which consider numerous host taxa at *Marteilia*-positive sites have sometimes found that not all viable host types are infected, or that a particular host taxon can display advanced disease caused by the ‘unexpected’ species (e.g. Le Roux *et al.* 2001; Novoa *et al.* 2005; Arzul *et al.* 2014). Arzul *et al.* (2014) highlight the hypothesis that when environmental parasite loads are high a predominant type could infect both oysters and mussels (Carrasco *et al.* 2007*a*,*b*), and possibly other hosts/vectors. Further, although both *Marteilia* species can infect multiple hosts, a controlled study is required to test the hypothesis that there is a significant difference in host preference of *M. refringens* for oysters and *M. pararefringens* for mussels. However, we do not use this hypothesis as part of our justification for separating the two species. Notably, *M. pararefringens* has not been detected in any other invertebrates coexisting with infected mussels in the UK, despite extensive PCR-screening of other molluscs, crustaceans and polychaetes and other invertebrates using the new *Marteilia*-specific primers developed in this study, and paramyxid-specific primers (Ward *et al*. 2016; plus unpublished data) across samples collected between 2013 and 2017. Its apparent restriction to *M. edulis* as a host in northern Europe may similarly be due to currently unknown ecological/lifecycle factors, or/and competition with other parasites. It is interesting that screening of environmental samples (water, sediment) from near bivalve beds in the Tamar Estuary did not detect *M. pararefringens* at the same sampling time that no sampled individuals were found to be positive by histology or specific PCR (2017). Current research seeks to determine whether levels of detection by eDNA screening correlate with levels of infection in proximally situated hosts. None of the potential benthic alternate hosts we screened were *M. pararefringens*-positive, suggesting that the *M. pararefringens* eDNA signal we detected derived from either zooplanktonic vectors (e.g. Arzul *et al*. 2014) or freely occurring material (e.g. spores).

The combined molecular phylogenetic and biogeographical evidence is more than sufficient to consider the O- and M-type lineages (in any case a misleading terminology) as separate species. We, therefore, amend the existing diagnosis for *M. refringens* to include the diagnostic five invariant ITS1 sequence positions shown in Fig. 2B, and erect a new species, *Marteilia pararefringens* n. sp., distinguished from *M. refringens* by (1) clear and consistent molecular signatures unique to each species across the rRNA gene array; (2) strongly supported, mutually exclusive phylogenetic clustering of the two species when multiple gene regions are used for the analyses; and (3) different geographical distributions, indicated by non-detection of *M. refringens* in areas sampled in northern Europe (tissue samples from oysters and mussels in the UK, Norway, Sweden and other invertebrates, and environmental samples in the UK), whereas *M. pararefringens* is found in most of those sample types, even if often at low levels.

Further work should more precisely describe the eco-pathological and biogeographical differences between these two species, and also investigate the significance of the marked variation in ITS1 and IGS regions within both species (Figs 2 & 3). We hypothesize that *M. refringens* is more temperature-sensitive than *M. pararefringens*, which constrains the latitudinal range and/or virulence of the former under certain conditions, while *M. pararefringens* is more ecologically tolerant, geographically widespread and can cause disease over a large part of its range. Both species show peak infection levels and prevalence during the summer months (in all parts of their ranges), with additional peaks possible in spring. Different sites can show different seasonal patterns (Ifremer; EU Reference Laboratory for Molluscan Diseases). Therefore, on the basis of existing knowledge, seasonality does not appear to differ between the two species. Distribution may also be impacted by range/behavioural optima for intermediate hosts (van Banning, 1979) such as the copepod *Paracartia* (Arzul *et al.* 2014).

Longshaw *et al*. (2001) concluded that *M. refringens* and *M. maurini* (now *M. pararefringens*) could not reliably be separated using ultrastructural criteria. However, this conclusion was reached on the basis of examination of only 20 individuals: 14 infected *M. edulis* and six infected *O. edulis*, on the assumption that the former infections were of *M. maurini* and the latter *M. refringens* (Longshaw *et al*. 2001). Even if that assumption was correct, these numbers are very likely too low to detect subtle ultrastructural differences between the lineages since it would be necessary to compare directly the ultrastructure of equivalent developmental/maturation stages of the parasites (Longshaw *et al*. 2001). If such differences do exist it is possible that far larger numbers of (genetically typed) individuals of each species would be required to statistically confirm them. It is unlikely that resources for a study of such scale would be prioritized, at least before detailed genomic analyses had identified the full extent and nature of the evolutionary and functional differences between the species.

A final important point to emphasize is that the relative potential of both species to cause epidemics or mass mortalities in any part of their ranges remain very poorly known. Our literature survey highlighted that in many reports of disease events the genotype of the causative agent(s) is not (clearly) reported. Accurate identification of *M. refringens* and *M. pararefringens* has very significant implications for the trading of host species susceptible to these pathogens. If (as at present) they are considered as a single entity under EC Directive 2006/88, and by the OIE (2017), areas from which only one species has been detected are open to the importation of hosts originating from locations where the other species is known to occur. As so little is known of the pathogenicity of both species under different sets of biotic and abiotic conditions, indiscriminate human-mediated distribution of them, or their close relatives (Ward *et al*. 2016), is biologically inadvisable. In essence, the application of high resolution systematics of the kind demonstrated in this study has the potential to allow refinement of the listing of important pathogens such as *Marteilia* and to ensure that further spread does not occur between infected areas and those currently free of certain types. Integration of data pertaining to biogeography, host and pathogen ecology (including alternative hosts, vectors, seasonality, etc.), and molecular systematics should be consistently applied to facilitate this process (Stentiford *et al.* 2014). We propose that the data presented in this study should facilitate updating of the listing of *M. refringens* in both OIE and EC legislation.

**Nomenclatural acts:**

Class Ascetosporea Sprague, 1979 stat. nov. Cavalier-Smith, 2002 emend.

Order Paramyxida Chatton, 1911

Genus Marteilia Grizel et al, 1974

*Marteilia refringens* Grizel et al, 1974. Revised diagnosis: As for *M. refringens* Grizel et al, 1974, with the addition of ITS1 type sequence DQ426611 (EURL *M. refringens* ‘O-type’ reference sequence), containing the five diagnostic positions emboldened and underlined in the following (the first position corresponding to position 321 of DQ426550): G**T**CAGGCGAGTGCTCTCGTTGC**C**CTT**T**CCC**CG**ACGGCCGTG.

*Marteilia pararefringens* n. sp. Bass, Stentiford and Kerr, 2017.

Diagnosis: As for *M. refringens* Grizel et al, 1974, with the addition of ITS1 type sequence DQ426550 (EURL *M. refringens* ‘M-type’ reference sequence), containing the five diagnostic positions emboldened and underlined in the following (the first position corresponding to position 321 of DQ426550): G**C**CAGGCGAGTGCTCTCGTTGC**G**CTT**G**CCC**T-**ACGGCCGTG.

## Supplementary material

For supplementary material accompanying this paper visit http://dx.doi.org/10.1017/S003118201800063X. click here to view supplementary material
