# Supplementary material for: Marteilia refringens and Marteilia pararefringens sp. nov. are distinct parasites of bivalves and have different European distributions
Source: Parasitology. 2018 Jun 11;145(11):1483–92. doi: 10.1017/S003118201800063X (PMC6137380; doi:10.1017/S003118201800063X)
Supplement: Supplementary file 1 [file S003118201800063Xsup001.pdf]

Supplementary Table 1. PCR primers used to generate coverage of *M. refringens* isolates across the full rRNA gene array, and the new *M. refringens*-specific ITS1 nested primer set.

Name	Sequence 5'-3'	Product size bp	Annealing temp	Region amplified
12F	GGTATTGAACGAACGGACG	927	53	End of ITS2
12R	CATGGCCTTCACTCAGCG			start of 28S
13F	TCTGTCGAAACTCGCGAGG	852	53	End of ITS 1
13R	GTGGGTAGTCGCGCACG			Start of ITS2
14F	CACACGTCATACAATGCCCG	1061	57	End of 18s
14R	AGAAGGACGCAGCAATCTGC			Start of 5.8s
24F	AGGCGAGTGCTCTCGTTGC	393	57	Start in ITS1
24R	GATCGTACGATCACGATGCG			Start of ITS2
25F	TGGTCGTCGTAGCGCTCG	495	55	Start of ITS2
25R	ATCTAATGGCCATCGTTGGC			End of ITS2
26F	ATCTAACGCGCGAGTCGCG	519	57	End of ITS2
26R	TGTGACGTCGAGACGAGCG			start of 28s
MartDBITSf1:	CTCGTGGAGCGGGTCTACCG			V9 region of 18s
MartDBITSr1:	TATCACGCCGCTGAATGCTTTCG	1220bp	65	
MartDBITSf2:	TACGCCGTGCCGAGAGGTAC			
MartDBITSr2:	GCGTCCTTCTACGGCTCACGATC	1034bp	65	5.8s

Supplementary Table 2. ITS1 rDNA *Marteilia refringens* sequences from GenBank

**Reference**	**Country**	**Host**	**Accession and Type**	**Disease status**	**Methods used /Gene amplified**
López-Sanmartín et al 2015	Southern Portugal and Spain	*Ostrea edulis* (Portugal)<br><br>*Ostrea stentina* (Spain and Portugal)	KR149574 **(O)**<br><br>KR149573 **(M)**<br><br>KR149572 **(M)**<br><br>KR149571 **(O)**<br><br>KR149570 **(O)**	Found (O and M) in Portugal in *O.edulis* and *O. stentina* respectively. Found (M) in Spain in *O. stentina.*<br><br>Found during screening for *Marteilia*. First detection in Portugal.	Histology, in situ hybridisation ITS (PR4-PR5; Le Roux et al 2001)
Elgharsalli et al 2013	Monastir Bay, Tunisia	*Ostrea stentina*<br><br>Dwarf oyster<br><br>103 specimens 2009,<br><br>20 specimens 2010	5 x ITS samples sequenced 2009 and 1 x sample from 2010<br><br>JX119018 **(O)**<br><br>JX119019, JX119022 **(O)**<br><br>JX119020-21 **(O)**	Site previously linked to unknown mass mortalities in 2007, samples collected after this occurrence in 2009-2010.	ITS (PR4-PR5; Le Roux et al 2001) PCR, RFLP, cloned and sequenced. (85/103 +ve ITS PCR)
Balseiro et al 2007	La Spezia, Italy (18)<br><br>Thessaloniki,	*Mytilus galloprovincialis*<br><br>*Mytilus edulis*<br><br>Cultured mussels<br><br>*Ostrea edulis*	DQ426546, 557-560, 563, 565, 570-574, 586, 588, 589, 590-591, 593, 595, 597, 598, 602-604 **(O)**<br><br>DQ426569, 576-578 **(O)**<br><br>DQ426545, 547, 548, 549, 556, 561, 562, 567, 579, 580, 582, 584, 592, 594,<br><br>599,600,601 **(M)**<br><br>DQ426550-555, 566, 568, 575, 581, 585 **(M)**<br><br>DQ426583, 587, 596 **(O)**	Thessaloniki has been positive for *Marteilia* by histology. (Virvilis et al 2003 no PCR completed)	ITS (PR4-PR5; Le Roux et al 2001) PCR, RFLP, cloned and sequenced.

	Greece (2)<br><br>Kavala, Greece (28)	Wild oysters at both locations.	DQ426564 **(O)**<br><br>DQ426608-613, 615-620, 623, 626-636, 639-640, 642 **(O)**<br><br>DQ426624-625 **(O)**<br><br>Total DQ426545-DQ426644		
Arzul et al 2014	Corsica<br><br>Diana Lagoon	*Mytilus galloprovincialis*<br><br>Zooplankton:<br><br>Copepods, (*Paracartia latisetosa)*, Cladocera, Appendicularia, Chaetognatha, Polychaeta	(5 clones from 3 samples) KC462432-33 **(M)**<br><br>(3 clones from 3 samples)<br><br>KC462434-435 **(O)**<br><br>KC462430-31 **(M)**<br><br>KC462436-37 **(*Marteilia cochillia*)**	Endemic site for Marteiliosis in mussels *M.galloprovincialis* and *O.edulis*	ITS (PR4-PR5; Le Roux et al 2001) PCR, RFLP, cloned and sequenced.<br><br>51 mussels M profile / 2 mussels had both M and O profiles)
Gombac et al 2014	Slovenia Adriatic Sea	*Mytilus galloprovincialis*<br><br>Mussel farm (960 cultured mussels)<br><br>Natural mussel beds (320 wild mussels)	JQ898012<br><br>JQ898013<br><br>JQ898014 **(M)**	First detection of *M.refringens* in *M.galloprovincialis* in this location. Only 4 of 1280 positive for *Marteilia* but infection was quite severe. All 4 from cultured mussels.	ITS (PR4-PR5; Le Roux et al 2001) PCR, cloned, RFLP and sequenced.<br><br>The one type O restriction profile grouped with type M when sequenced.

Novoa et al 2005	Huelva SW Spain<br><br><br><br><br><br><br><br><br><br><br><br>Ria de Arosa NW Spain<br><br><br><br>Ria de Vigo NW Spain<br><br><br><br><br><br><br><br><br><br><br><br>Delta de Ebro	*Ostrea edulis*<br><br><br><br><br><br><br><br><br><br><br><br>*Mytilus galloprovincialis*<br><br><br>*Mytilus galloprovincialis*<br><br><br><br><br><br><br><br><br><br><br><br><br>*Ostrea edulis*	AY324558, **(M)**<br><br>AY324562, 564, 567, 568 **(M)**<br><br>AY324586 **(O)**<br><br>AY324581, 577, 576, 588 **(O)**<br><br>AY324578 **(O)**<br><br><br>AY324556 **(M)**<br><br><br>AY324565 **(M)**<br><br>AY324551-555, 559-561, 563, 569-573 **(M)**<br><br>AY324584, 582 **(O)**<br><br>AY324574 **(O)**<br><br><br>AY3234557 **(M)**<br><br>AY324575, 579, 580, 583, 587 **(O)**<br><br><br><br><br>Total AY324551-AY324588	Endemic zones for marteiliosis	ITS (PR4-PR5; Le Roux et al 2001) PCR, RFLP, cloned and sequenced.
López-Flores et al 2004	Huelva, Spain<br><br><br><br><br><br>Huelva, Vigo, (Spain)<br><br><br><br><br><br><br><br><br>Trieste, Venice (Italy)	*Ostrea edulis*<br><br><br><br><br><br>*Mytilus galloprovincialis*<br><br><br><br><br><br><br><br><br>*Mytilus galloprovincialis*	AJ629336 **(O)**<br><br>AJ629334-5 **(O)**<br><br><br><br>AJ629339-340 **(M)**<br><br>AJ629341-344 **(O)**<br><br>AJ629338 **(O)**<br><br><br>AJ629345 **(O)**<br><br>AJ629346-51 **(O)**<br><br><br>Total AJ629334-	Naturally infected oysters<br><br><br><br>Infected mussels	ITS (PR4-PR5; Le Roux et al 2001) PCR, cloned and sequenced.

			6, AJ629338-351		
Carella et al 2010	Tirrenian sea, south of Italy.	*Mytilus galloprovincialis*<br><br>Natural mussel beds and 14 mussel farms.<br><br>30 animals from each site.	Bagnoli AB534169 **(M)**<br><br>Salerno AB513427 **(M)**<br><br>Naples AB534170 **(M)**	Natural bed of *Mytilus* had a 15% presence of *Marteilia*<br><br>2/14 mussel farms had a 14% and 25% presence of *Marteilia*<br><br>Part of a monitoring project to assess animal health status.	ITS (PR4-PR5; Le Roux et al 2001) PCR, RFLP, cloned and sequenced.
Le Roux et al 2001	La Trinite, France<br><br>Marennes, France<br><br>Leucate, France<br><br>Vigo, Spain<br><br>Istria, Croatia	*Mytilus edulis*<br><br>*Ostrea edulis* both from both geographic locations<br><br>*Mytilus galloprovincialis*<br><br>*Ostrea edulis* both from all geographic locations	La Trinite *M.edulis* 15M/1O<br><br>La Trinite *O.edulis* 1M/20O<br><br>Marennes *M.edulis* 0M/0O<br><br>Marennes *O.edulis* 0M/15O<br><br>Leucate *M.gallo* 0M/0O<br><br>Leucate *O.edulis* 0M/16O<br><br>Vigo *M.gallo* 4M/0O<br><br>Vigo *O.edulis* 0M/0O<br><br>Istria *M.gallo* 7M/0O<br><br>Istria *O.edulis* 0M/0O<br><br>**Sequences not on GenBank**	All naturally infected areas	ITS designed primers PR4-PR5 and RFLP on 2 infected animals from each area / cloned and sequenced.
Carrasco et al 2007a	Delta de l'Ebre, Spain<br><br>Alfacs and Fangar bays.	Zooplankton sampled<br><br>Cyclopoida *Oithona* sp and Harpaticoida sp two new hosts for *Marteilia*.	Positive results from PCR apparent for ITS (RFLP) and IGS PCRS but difficulty in separating individual zooplankton resulted in no sequencing being	Natural enzootic area of Marteiliosis previously site of Mussel farm and mortalities	ITS (PR4-PR5; Le Roux et al 2001) PCR, RFLP, sequenced.

			completed.		

Supplementary Table 3. IGS rDNA *Marteilia refringens* sequences from GenBank

Reference	Country	Host	Accession / Type	Disease status	Methods used /Gene amplified
Grijalva-Chon et al 2015	Gulf of California, Mexico	*Crassostrea gigas*<br><br>*Crassostrea corteziensis*	JQ066723-JQ066726 **(Type O)**	Previous massive mortalities 1990-2009	IGS (MT1/MT2, MT-1B /MT-2B; López-Flores et al 2004) nested PCR and sequenced.
López-Flores et al 2004	Huelva, Spain	*Ostrea edulis*	AJ604561 **(Type O)**<br><br>AJ629353-356, 370, 374-375 **(Type O)**<br><br>AJ629352, 357-369, 371-373, 376 **(Type M)**	Naturally infected areas	IGS (MT1/MT2, MT-1B /MT-2B; López-Flores et al 2004) nested PCR, cloned and sequenced.
Carrasco et al 2007b	Alfacs Bay, Delta de L'Ebre, Catalonia Spain	*Mytilus galloprovincialis* (60)<br><br>Zooplankton	AM504146-149 **(Type M)**<br><br>AM504129-145 **(Type M)**	Alfacs Bay is an endemic area for *Marteilia* parasite. Details of individual *Mytilus* infection intensity through year.	IGS (MT1/MT2, MT-1B /MT-2B; López-Flores et al 2004) nested PCR, sequenced.
López-Flores et al 2008a	Bay of Palma, Mallorca, Balearic Islands (Spain)	*Chamelea gallina* Striped clam	AM292652 **(Type O)**<br><br>from 3 clams	Histopathological survey in response to mass mortality of clams but no clear association between mortality and *Marteilia* found as only 3 of 69 clams infected.	IGS (MT1/MT2, MT-1B /MT-2B; López-Flores et al 2004) nested PCR, sequenced and *in situ* hybridisation confirmed presence in digestive gland tissue.
López-Flores et al 2008b	Huelva, SW Spain	*Solen marginatus*<br><br>Razor clam	AM748037-043 **(Type M)**<br><br>(5 of the 9 samples positive by PCR)	*Marteilia* previously suspected in razor clam at this site so more sampling was done for this study.	IGS (MT1/MT2, MT-1B /MT-2B; López-Flores et al 2004) nested PCR, sequenced and *in situ* hybridisation confirmed presence in digestive gland tissue and phylogenetic analysis.

Pascual et al 2010	Ria de Vigo, Spain.	*Xenostrobus securis*<br><br>5 out of 30 samples	EU854303-307	Histopathological analysis of individuals showed presence of *Marteilia*, confirmed by PCR. The pathological conditions found in *X.securis* were previously found at same site in *M.galloprovincialis*.	IGS (MT1/MT2, MT-1B /MT-2B; López-Flores et al 2004) nested PCR, cloned and sequenced.
Elgharsalli et al 2013	Monastir Bay, Tunisia	*Ostrea stentina*<br><br>Dwarf oyster<br><br>103 specimens 2009<br><br>20 specimens 2010	2 x IGS samples sequenced (2009) and 1 sample IGS from 2010.<br><br>100% match to AJ629355<br><br>**Sequences not on GenBank**	Site previously linked to unknown mass mortalities in 2007, samples collected after this occurrence in 2009-2010.	IGS (MT1/MT2, MT-1B /MT-2B; López-Flores et al 2004) nested PCR.
Boyer et al 2013	Sete Channel,<br><br>Thau Lagoon,<br><br>Southern France.	*M.galloprovincialis*<br><br>*R.decussatus*<br><br>*P.grani* (pooled samples)<br><br>One sample each month was positive for *M.refringens* by PCR	*R.decussatus*<br><br>100% homology to AM504148, AM748037, AM504140, AJ629359<br><br>For *P.grani* (pooled) PCR products sequenced and found to be 99% similar to<br><br>EU854304<br><br>AM748040<br><br>AJ629366<br><br>AJ629361<br><br>AJ629360<br><br>AJ629358<br><br>**Sequences not on GenBank**	Detected *M. refringens* in *O.edulis* 1970s now in *M.galloprovincialis* in 1985. 21.5% to 2.86% in 2006. In this study monitored the presence of the parasite for 1 year investigation of the *P.grani* in life cycle of *M.refringens*. Description of detection frequency of *M.refringens* throughout year.	IGS (MT1/MT2, MT-1B /MT-2B; López-Flores et al 2004) nested PCR, sequencing and *in situ*.
Carrasco et al 2007a	Delta de l'Ebre, Spain<br><br>Alfacs and Fangar bays.	Zooplankton sampled<br><br>Cyclopoida *Oithona* sp and Harpaticoida sp two new hosts for	Positive results from PCR apparent for ITS (RFLP) and IGS PCR but difficulty in separating individual zooplankton	Natural enzootic area for Marteiliosis, previously site of mussel farm and mortalities.	IGS (MT1/MT2, MT-1B /MT-2B; López-Flores et al 2004) nested PCR.

		Marteilia.	resulted in no sequencing being completed.		

Supplementary Table 4. Positions of sequence signatures corresponding to *M. refringens* O- and M-types, across the entire rRNA gene array. The fifth column indicates the number of independent samples available for determination of signatures. The five invariant ITS1 signatures are shown in bold.

Region	Position in Assembly	Type O signatures	Type M signatures	No. O/M	Notes
**ITS1**	1886	A	G	2/2	
	1929	A	G	2/2	
	1983	G	T	2/2	
	1985-7	-	GTC	2/2	
	**2334**	**T**	**C**	**99/76**	
	**2355**	**C**	**G**	**99/76**	
	**2359**	**T**		**99/76**	
	**2363**	**C**	**G**	**99/76**	
	**2364**	**G**	**C**	**99/76**	
	2432-4	-	ATC	3/2	
	2441	G	A	3/2	
**ITS2**	2636	A	G	3/2	R ambiguity in Spanish_mussel125
	2748	A	G	3/3	
	2842	G	A	3/3	
	2888-90	-	ACA	3/3	
	3018	G	A	3/3	
	3131	T	A	3/3	
	3372-3	*C*	*-*	*3/2*	
	3374	*A*	*G*	*3/2*	
	3379	*C*	*G*	*3/2*	
	3380	*C*	*A*	*3/2*	
	3381-2	*A*	*-*	*3/2*	
	3395	*A*	*C*	*3/2*	
	3396	*-*	*C*	*3/2*	
**LSU**	4783	T	C	5/8	Coverage insufficient; others very likely exist
**IGS**	8783-4	AA	TT	2/2	
	8797	A	G	2/2	
	8975	A	C	12/30	
	9098-9103	TC	-	14/52	C of insertion is reliable in all but 2 cases; IGS_SporeF2 = TC
	9191	C	T	12/52	Y ambiguity in 2 GenBank sequences
	9269	T	C	12/52	Y ambiguity in 2 GenBank sequences
	9473	G	C	5/3	Spanish_mussel125 = G
	9512	G	A	5/3	Spanish_mussel125 = G
